# Phosphodiesterases in non-neoplastic appearing colonic mucosa from patients with colorectal neoplasia

**DOI:** 10.1186/s12885-016-2980-z

**Published:** 2016-12-07

**Authors:** Badar Mahmood, Morten Matthiesen Bach Damm, Thorbjørn Søren Rønn Jensen, Marie Balslev Backe, Mattias Salling Dahllöf, Steen Seier Poulsen, Niels Bindslev, Mark Berner Hansen

**Affiliations:** 1 Digestive Disease Center K, Bispebjerg Hospital, Copenhagen, DK-2400 Denmark; 2 Department of Biomedical Sciences, Faculty of Health Sciences, University of Copenhagen, Copenhagen, DK-2200 Denmark; 3 Zealand Pharma, Glostrup, DK-2600 Denmark

**Keywords:** Endoscopic biopsy, Human, Cyclic nucleotide phosphodiesterases and colorectal cancer

## Abstract

**Background:**

Intracellular signaling through cyclic nucleotides, both cyclic AMP and cyclic GMP, is altered in colorectal cancer. Accordingly, it is hypothesized that an underlying mechanism for colorectal neoplasia involves altered function of phosphodiesterases (PDEs), which affects cyclic nucleotide degradation. Here we present an approach to evaluate the function of selected cyclic nucleotide-PDEs in colonic endoscopic biopsies from non-neoplastic appearing mucosa.

**Methods:**

Biopsies were obtained from patients with and without colorectal neoplasia. Activities of PDEs were characterized functionally by measurements of transepithelial ion transport and their expression and localization by employing real-time qPCR and immunohistochemistry.

**Results:**

In functional studies PDE subtype-4 displayed lower activity in colorectal neoplasia patients (*p* = 0.006). Furthermore, real-time qPCR analysis showed overexpression of subtype PDE4B (*p* = 0.002) and subtype PDE5A (*p* = 0.02) in colorectal neoplasia patients. Finally, immunohistochemistry for 7 PDE isozymes demonstrated the presence of all 7 isozymes, albeit with weak reactions, and with no differences in localization between colorectal neoplasia and control patients. Of note, quantification of PDE subtype immunostaining revealed a lower amount of PDE3A (*p* = 0.04) and a higher amount of PDE4B (*p* = 0.02) in samples from colorectal neoplasia patients.

**Conclusion:**

In conclusion, functional data indicated lower activity of PDE4 subtypes while expressional and abundance data indicated a higher expression of PDE4B in patients with colorectal neoplasia. We suggest that cyclic nucleotide-PDE4B is overexpressed as a malfunctioning protein in non-neoplastic appearing colonic mucosa from patients with colorectal neoplasia. If a predisposition of reduced PDE4B activity in colonic mucosa from colorectal neoplasia patients is substantiated further, this subtype could be a potential novel early diagnostic risk marker and may even be a target for future medical preventive treatment of colorectal cancer.

**Electronic supplementary material:**

The online version of this article (doi:10.1186/s12885-016-2980-z) contains supplementary material, which is available to authorized users.

## Background

Colorectal cancer (CRC) is a major global health problem and has one of the lowest survival rates for all types of cancers [1]. Colorectal neoplasia (CRN) is correlated with the development of CRC [2].

The pathogenesis and etiology of CRC and CRN are still not fully understood. With regards to CRC, non-steroidal anti-inflammatory drugs (NSAIDs) decrease mortality rate and inhibit *de novo* disease development. The exact mechanism by which NSAIDs exert their anticarcinogenic effect is not completely understood [3, 4]. Investigators attribute part of the anticancer activity of NSAIDs to their inhibition of the cyclooxygenase (COX) enzymes, leading to a decrease in activity of prostaglandins and cyclic adenosine monophosphate (cAMP) [5, 6].

Recently some COX-independent targets have been identified as being involved in the anticancer effects of NSAIDs [3, 7–10]. Moreover, both cAMP and cyclic guanosine monophosphate (cGMP) phosphodiesterases (PDEs) have gained particular interest in relation to CRN [3, 11, 12]. PDEs are metallophoshydrolases which specifically hydrolyzes the 3′,5′-cyclic phosphate moiety in cyclic nucleotides (cNTs) to a non-cyclic 5′ monophosphate, thereby deactivating cAMP and cGMP. PDE activity terminates second messenger signaling by degrading cNTs, whereas inhibition of PDE activity blocks cNT hydrolysis to mimic or amplify cNT signaling. The PDE superfamily consists of 20 distinct genes divided into 11 protein families, PDE1-11 [13].

Studies on human colon cancer cell lines show decreased levels of cGMP and elevated levels of PDE5 mRNA [3, 14], indicating a perturbed activity of the PDEs involved in degrading cGMP in CRC. Similar studies have been conducted investigating the role of cAMP in CRC [4]. However, studies in cancer cell lines, show conflicting outcomes regarding the role of prostaglandins, cAMP and cGMP in CRN and CRC pathogenesis [15–20].

In order to identify a potential predisposition in colonic mucosa for development of CRN in non-neoplastic colon, we decided to study the function, expression and localization of several PDE subtypes in specimens of endoscopically non-neoplastic appearing colonic mucosa.

## Methods

### Aim

The aim of this study was to investigate function, expression and localization/abundance of selected PDEs in biopsies from non-neoplastic appearing colonic mucosa obtained from patients with and without CRN. Thus, questioning if a possible predisposition to the cancer disease exists in non-neoplastic appearing colonic mucosa.

### Study population

Patients referred for a colonoscopy on suspicion of CRC, were included and divided into two groups. The first group consisted of patients with present or history of CRN, while the second group was control patients (i.e. CTRL) with no present or history of CRN. Patients with incomplete colonoscopy, hemorrhagic diathesis, and inflammatory bowel disease or with previous sigmoid resection were excluded from the study. A total of 27 subjects were enrolled, hereof 12 CRN-subjects (4 women) and 15 CTRLs (7 women). For each patient, we noted age, body mass index (BMI), previous illnesses, medication, all signs of earlier colorectal disease and the findings during the colonoscopy. Age and BMI were well balanced between groups. Mean age (±SEM) for CTRL patients was 58 ± 3.7 and for CRN patients 66 ± 3.7 years. Mean BMI was 24.9 ± 1.5 in CTRL patients and 26.2 ± 0.9 in CRN patients. Eight patients from the CTRL and 6 patients from the CRN group had comorbidities such as ischemic heart disease, heart failure, hypertension, atrial fibrillation, diabetes, kidney failure, chronic obstructive lung disease, dyslipidemia, intermittent claudication, peptic ulcer disease, osteoporosis, rheumatic arthritis, polycystic ovary syndrome and pacemaker implantation. There were no apparent differences between the two groups in comorbidity. Eight patients in the CTRL group and 8 patients in the CRN group were using regular medications e.g. anti-thrombotic, ACE inhibitors, statins, levothyroxine, proton pump inhibitors, angiotensin II receptor antagonists, glucocorticoids, β-blockers, β-agonists, anti-histamines, xanthine oxidase inhibitors, diuretics, antifolate and anti-emetics. There were no apparent differences between the two groups in prescribed medications. None of the drugs has a direct effect on the PDE metabolism.

### Ethics

The Scientific Ethical Committee of Copenhagen (H­3­2013­107) and The Danish Data Protection Agency approved the study protocol (BBH-2013-024, I-Suite no: 02342). The study was conducted by the Helsinki declaration. All patients participating gave written informed consent.

### Biopsy extraction and processing

Six biopsies from each patient were obtained during endoscopy from non-neoplastic appearing colonic mucosa using standard biopsy forceps (Boston Scientific, Radial Jaw 4, outside diameter of 2.2 mm). Biopsies were obtained approximately 30 cm orally from the anal verge and at least 10 cm from endoscopically abnormal tissue (i.e. neoplasia). The biopsies were immediately transferred to an iced, oxygenized bicarbonate Ringer solution with the following composition (in mM): Na^+^ (140), Cl^−^ (117), K^+^ (3.8), PO_4_^3−^ (2.0), Mg^2+^ (0.5), Ca^2+^ (1.0), glucose (5.5) and HCO_3_^−^ (25). Before obtaining biopsies, the media pH was adjusted to 7.4 by gassing with 95% O_2_/5% CO_2_.

### Chemicals

Theophylline, indomethacin, acetazolamide, zaprinast, cilostamide, rolipram, cAMP and cGMP were purchased from Sigma-Aldrich (Seelze, Germany). Sildenafil, db-cAMP, cGMP, and antibodies for PDE3B (cat. no.: SC-376823), PDE4A (cat. no.: SC-74428), PDE4B (cat. no.: SC-25812), PDE4D (cat. no.: SC-25097) and PDE5A (cat. no.: SC-32884) were purchased from Santa Cruz Biotechnology (Santa Cruz, CA, USA). Antibodies for PDE3A (cat. no.: LS-A732) and PDE4C (cat. no.: LS-C185777) were purchased from LifeSpan BioSciences, Inc. (Seattle, WA, USA). Primer sequences were synthesized by TAGCopenhagen (Copenhagen, Denmark). The human PDE10 real-time PCR primers were purchased from Qiagen. Bumetanide was obtained from Leo Pharmaceuticals (Copenhagen, Denmark). All other chemicals were of analytical grade.

### Pretreatment

Up to 4 biopsies from each patient were mounted in Mini-Ussing-Air-Suction-Chambers (MUAS chambers) for measuring transepithelial electrolyte transport captured as short circuit currents (SCC). The method used has previously been described in detail [21, 22]. Correction for media resistance between tips of potential electrodes was carried out just before mounting of biopsies, thus circumventing challenges of edge damage in measuring correct active ion transport. The time between the biopsy procedure and mounting of up to 4 biopsies into MUAS chambers was about 45 min during which the samples were kept in oxygenated-Ringer solution at approximately 0 °C. Following approximately 15 min of equilibration, baseline SCC and slope conductance values were recorded. Amiloride (20 μM) was subsequently added to the luminal side of the MUAS chamber to inhibit epithelial sodium channels and thereby reducing cell energy expenditure. Indomethacin (13 μM) was added to the basolateral side to inhibit endogenous synthesis of cNTs. Experiments were resumed after an additional 20 min equilibration period. If stable SCC was not achieved during pretreatment, the biopsy was excluded from further study.

### Cyclic-AMP-related PDEs and inhibitors

In one part of our functional study, db-cAMP (16 μM) was applied on the basolateral side of biopsies. After 10 min, biopsies were then exposed to cilostamide (PDE3 inhibitor, 1 μM) or rolipram (PDE4 inhibitor, 1 μM). PDE3 and PDE4 primarily degrade cAMP [23, 24], and their inhibition elicited a rise in SCC. Then a dose of theophylline (400 μM, added bilaterally), a competitive and nonselective PDE-inhibitor raising intracellular levels of cNT [25], was applied to give maximal inhibition of PDE activity and maximal cNT-induced SCC. Finally each experiment was terminated after adding bumetanide (25 μM), ouabain (200 μM) and acetazolamide (250 μM), alone or in combination to the basolateral side of the biopsies.

### Cyclic-GMP investigation

In a second part of the functional study, cGMP (16 μM) was applied to biopsies. Previous reports have provided data showing more consistent responses when cGMP is applied vs db-cGMP [21]. After 10 min, samples were exposed to sildenafil (1 μM) or zaprinast (1 μM), both PDE5 and PDE10 inhibitors [26]. However, when no changes in SCC were observed, concentrations were increased stepwise to reach a final concentration of maximally 500 μM for the inhibitors and 50 μM for cGMP. Each experiment was terminated following the basolateral addition of bumetanide (25 μM), ouabain (200 μM) and acetazolamide (250 μM), alone or in combination.

### RNA extraction from biopsies

One biopsy from each patient was obtained for RNA-analysis. The mean wet weight of each biopsy was 5.8 mg. Thus we were only able to perform qPCR on a selected number of PDE isoforms. Biopsies were immediately transferred to RNA Later (Life Technologies, Naerum, Denmark). Biopsies were homogenized using a TissueLyser II (Qiagen, Copenhagen, Denmark), and RNA was extracted using NucleoSpin RNA® (Macherey-Nagel, Düren, Germany). RNA concentration and purity were determined using a NanoDrop® ND-1000 (NanoDrop Technologies, Wilmington, DE, USA), and the latter as the A_260_/A_280_ and A_260_/A_230_ absorbance ratios.

### cDNA synthesis

RNA was converted to cDNA using the iScript™ cDNA Synthesis Kit (BioRad, Copenhagen, Denmark) according to the manufacturers protocol.

### Primer design

Primers against genes of interest and against β-actin [21] were designed using Primer3 (http://frodo.wi.mit.edu/primer3/input.htm) based on sequences obtained from Ensembl (http://www.ensembl.org/). The primer sequences were synthesized by TAGCopenhagen (Copenhagen, Denmark): **PDE3A** forward (5′-CAGAGTGAATCCCGTCACTTC-3′) reverse (5′-TGGTCCAAGTGGAAGAAACTC-3′); **PDE3B** forward (5′-TATGATCACCCAGGGAGGAC-3′) reverse (5′-GTTGTATTCTGGGCGAGAAAG-3′); **PDE4A** forward (5′-TGAAGACCGATCAAGAAGAGC-3′) reverse (5′-GTAATCCGACACGCAAAAGAT-3′); **PDE4B** forward (5′-ATCTCACGCTTTGGAGTCAAC-3′) reverse (5′-TTAAGACCCCATTTGTTCAGG-3′); **PDE4C** forward (5′-GCGATATCTTCCAGAACCTCA-3′) reverse (5′-CTTGTCACCTTCTTGGTCTCC-3′); **PDE4D** forward (5′-TTTCACGGTGGCACATACAT-3′) reverse (5′-GTGGACAAAATTTGCTTGGAG-3′); **PDE5A** forward (5′-TTGTGCAGAACTTCCAGATGA-3′) reverse (5′-TTTAGAGCAGCAAACATGCAC-3′); **β-Actin** forward (5′-ACCCAGCACAATGAAGATCA-3′) reverse (5′-CGTCATACTCCTGCTTGCTG-3′).

Dilution series of cDNA from HEK293 cells were run to verify acceptable amplification efficiencies and specificities by standard and dissociation curves for all primer sets.

### qPCR analysis

cDNA was amplified on a 7900HT Fast Real-Time PCR System (Applied Biosystems, Foster City, CA, USA) using Fast SYBR® Green Master Mix (Applied Biosystems) by the manufacturer’s manual. All samples were run in triplicates with β-actin primers on all plates. Results were analysed using SDS 2.3 (Applied Biosystems), and expression was calculated by the 2-ΔCT method.

### Immunohistochemical localization and quantification

One colon biopsy from each patient was put aside in 4% paraformaldehyde in 0.1 M phosphate buffer (pH 7.4), embedded in paraffin, and cut into 4 μm sections. Sections were boiled in citric buffer (pH 6 or 9) in a microwave oven for 15 min, followed by preincubation in 2% BSA for 10 min, and overnight incubation at 4 °C with primary antibodies. Subsequently, the sections were incubated for 40 min with biotinylated secondary antibody immunoglobulins, followed by a preformed avidin and biotinylated horseradish peroxidase macromolecular complex (code number PK-4000; Vector Laboratories) for 30 min. Three percent hydrogen peroxide was added after the second antibody layer. Finally, 3,3-diaminobenzidine (KEM-EN-TEC, catalog number 4170) was added for 15 min, followed by a 2 min incubation in 0.5% copper sulfate (Merck; catalog number 2790) diluted in Tris buffer containing 0.05% Tween 20. Counterstaining was performed with Mayer’s hemalun.

Images for quantification were recorded using a Zeiss Axioplan 2 plus microscope (Jena, Germany) fitted with a Photometrics CoolSNAP camera (Tucson, AZ, USA) and analysis was performed using Image-Pro Plus 7.0 software. A quantification of coloring was performed by to blinded investigators as a marker for protein abundance in biopsies (N_ctrl_ = 7, N_CRN_ = 8). Images for quantification were recorded at 20x magnification and the area measured represented 176000 μm^2^ of tissue. The area of stained structures was measured by selecting a colored region of interest. Automatically, areas with same color were measured - one image from each biopsy was measured. Mean ± SEM for samples in each group were calculated. Images for localization were recorded using a Zeiss Axio10 Imager A1 microscope (Jena, Germany) fitted with a Zeiss AxioCam ICc 3 camera (Jena, Germany) and analysis was performed using Image-Pro 9.1 software. Only mucosal layers were analyzed.

### Statistical analysis

Values are expressed as the mean ± SEM. The mean value was calculated and used when identical experiments were performed on several biopsies from the same patient. The statistical significance of differences between two groups was tested using Student’s *t*-test, provided that the variances of the groups were similar. For functional data, the Mann–Whitney *U*-test was applied in the case of unequal variance. For qPCR data, data with unequal variance were log transformed. When the variance of log transformed data was unequal, the Mann–Whitney *U*-test was applied. In qPCR experiments, when calculating statistics for multiple groups, one-way ANOVA with Tukey’s multiple comparisons test was used. A *p-*value less than 0.05 were considered statistically significant. Statistical analysis was done using SigmaPlot 12.3 for Windows, Systat Software Inc. (USA/Canada).

## Results

### Functional characterization of PDE activity

**Fig. 1:**
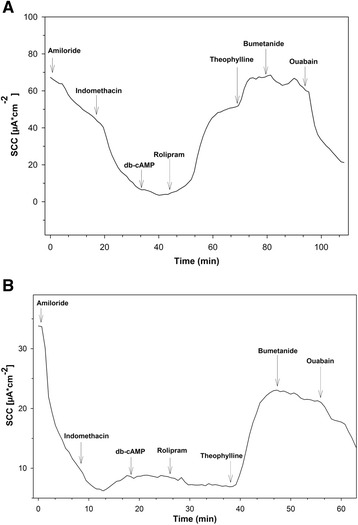
Short circuit current in biopsies from a control patient (**a**) and a CRN patient (**b**). The rolipram-induced increase in SCC observed in CTRLs (**a**) is not observed in CRN-patients (**b**). Biopsies mounted in the MUAS chambers were exposed to: amiloride (20 μM), indomethacin (13 μM), theophylline (100 μM), bumetanide (25 μM) and ouabain (200 μM)

**Fig. 2:**
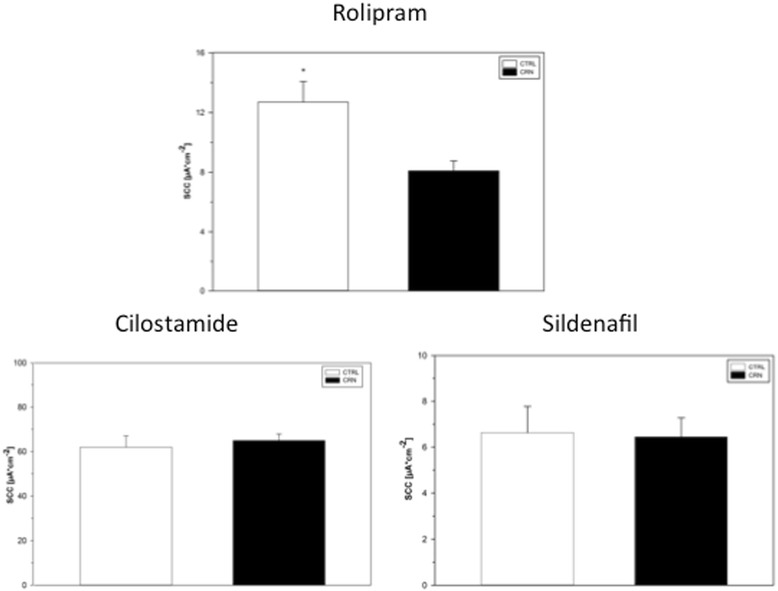
PDE inhibitor induced increase in SCC. Rolipram gave a significantly larger inhibition in CTRL- compared to CRN-patients, whereas, sildenafil and cilostamide showed no significant difference. Control patients: rolipram 12.7 ± 1.4 (*N* = 11, *n* = 11), sildenafil 6.6 ± 1.2 (*N* = 9, *n* = 9) cilostamide 64.9 ± 3.0 (*N* = 11, *n* = 13). Neoplasia patients: rolipram 8.1 ± 0.7 (*N* = 10, *n* = 10), sildenafil 6.4 ± 0.8 (*N* = 8, *n* = 8), cilostamide 61.9 ± 5.1 (*N* = 11, *n* = 12). Changes presented as mean SCC ± SEM. * Indicates a statistical difference. *p < 0.05

**Fig. 3:**
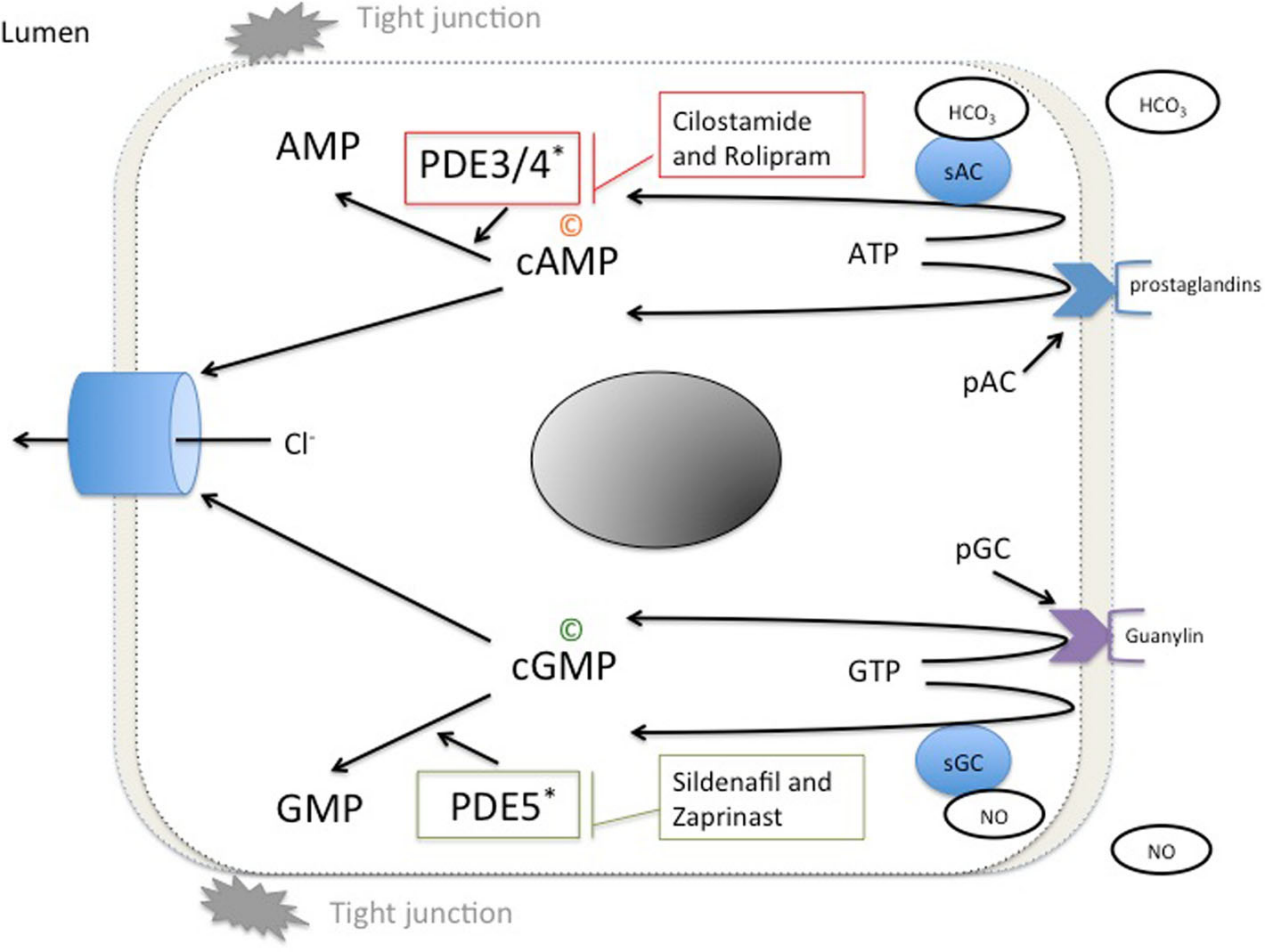
Basic mechanism of cyclic nucleotide regulation and function. This cartoon shows basic synthetic and regulatory pathways for cAMP and cGMP. P indicates decreased levels in colorectal neoplasia and C marks increased levels in colorectal neoplasia. *Enzymes with perturbed function in colorectal neoplasia. pGC, particulate guanylyl cyclase; pAC, particulate adenylyl cyclase; sGC, soluble guanylyl cyclase; sAC, soluble adenylyl cyclase; NO, nitric oxide; HCO_3_, bicarbonate; PDE, phosphodiesterase; Cl^−^, chloride ion

CTRL patients responded to rolipram (1 μM) with higher SCC increases compared to CRN patients (*p* = 0.006) (Figs. 1 and 2). This increase in SCC was 64% higher in CTRL patients (Fig. 2). No significant change was observed in SCC when samples, after prestimulation with db-cAMP, were exposed to either cilostamide (1 μM), (*p* = 0.869) or sildenafil (1 μM), (*p* = 0.899). No changes in SCC were recorded at even the highest concentrations of sildenafil or zaprinast. All biopsies presented an increase in SCC when exposed to theophylline. A decrease was observed when bumetanide and oubain were applied at the end of experiments, verifying the viability of samples to elicit a response. Up to 4 biopsies from each patient were successfully mounted in the MUAS chambers. At baseline mean SCC (±SEM) for CTRL and CRN patients was 68.2 ± 12.6 μA/cm^2^ and 78.2 ± 24.9 μA/cm^2^. During the observation period, slope conductance ranged between 36 and 150 mS⋅cm^−2^. To better understand the functional connection between SCC, PDE activity and cNT activity and inhibition, we present a simplified model (Fig. 3).

### PDE expression

**Fig. 4:**
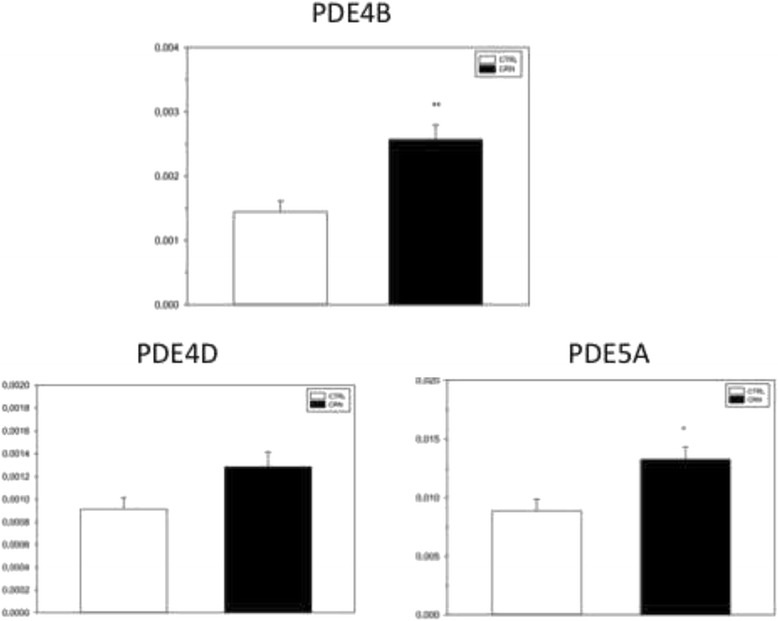
Expression levels for PDEs. Expression of subtypes PDE4B and PDE5A was significantly higher in the CRN patient group. For controls in PDE5A (*N* = 4, *n* = 4), PDE4B (*N* = 5, *n* = 5), PDE4D (*N* = 5, *n* = 5). For neoplasia in PDE5A (*N* = 5, *n* = 5), PDE4B (*N* = 8, *n* = 8), PDE4D (*N* = 8, *n* = 8). Expression levels are relative to ß-actin. Data presented as means ± SEM. *p < 0.05 and **p < 0.01

All 8 investigated PDEs were detected in biopsies by qPCR. Normalized to ß-actin levels, a significantly higher expression was measured for PDE4B and PDE5A in biopsies from CRN patients compared with CTRLs (Fig. 4). Expression level of PDE4B was 78% higher in CRN patients (*p* = 0.002), and PDE5A had a 49% higher expression level in the same group (*p* = 0.02). PDE3A had an expression level more than 10 fold greater than all other investigated PDEs (*p* < 0.0001), both in CRN and CTRL patients (Fig. 4). The expression of PDE4D showed a tendency of higher expression in CRN patients (*p* = 0.0712) (Fig. 4). No differences in expression levels of PDE10A were measured when comparing normal colonic mucosa from both CRN and CTRL patients (*p* = 0.6), the generated dataset is available as Additional file 1.

### Immunohistochemistry

**Fig. 5:**
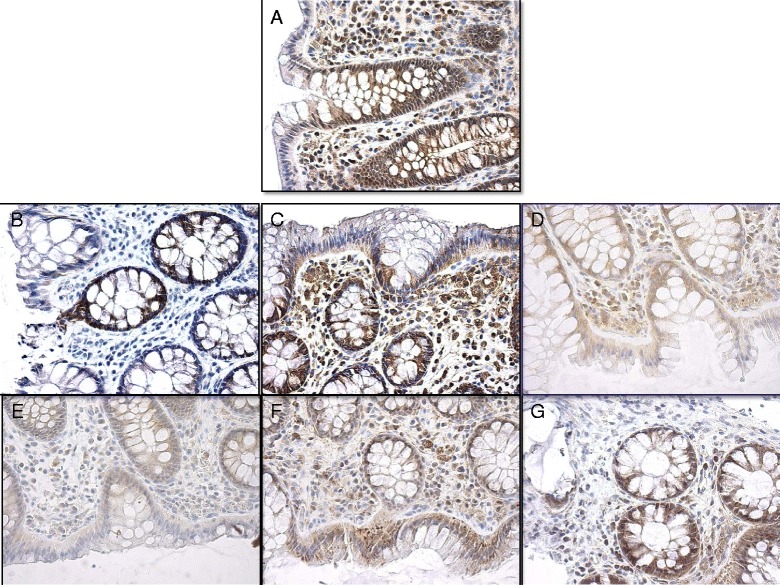
Immunohistochemical staining of endoscopic biopsy. Staining for A, PDE3A; B, PDE4A; C, PDE3B; D, PDE4C; E, PDE4B; F, PDE4D; G, PDE5A. All images are acquired at objective magnification of 40x

Examination of PDE3A-B, PDE4A-B-C-D and PDE5A in 15 biopsies was conducted at different tissue sections for each PDE. Consistent with all tissues sections and all examined biopsies, *N* = 7 for CTRL patients and *N* = 8 for CRN patients, our investigation revealed that all 7 PDE subtypes are present in normal appearing human sigmoidal mucosa. Figure 5 shows examples of biopsy specimens as measured by immunohistochemical staining with antibodies. The distribution of the isoenzymes locations varied. The PDE3A-antibody stained both epithelial and underlying cells in connective tissue, in a manner that indicated a non-specific reaction. Staining for PDE4B was specific for epithelial cells, while its localization at a subcellular level could not be determined.

Blinded quantification of specific coloring in immunohistochemical biopsies revealed a difference between CTRL patients and CRN patients for PDE3A, 11 403 ± 1209 vs. 8313 ± 762 (*p* = 0.035), and for PDE4B, 4276 ± 640 vs. 6723 ± 724 (*p* = 0.019), respectively. No difference was observed for PDE3B, PDE4A, PDE4C, PDE4D and PDE5A (Table 1). All included biopsy specimens predominantly contained epithelial cells and underlying connective tissue. In specific, no smooth muscle cells were observed.

**Table 1:** Immunohistochemical quantification data

PDE subtype	Sample Size, CTRL vs CRN	Mean (±SEM) CTRL vs CRN	*p*-value
PDE3A	14 vs 16	11404 (1209) vs 8313 (762)	0.035
PDE3B	14 vs 16	4098 (829) vs 4200 (908)	0.936
PDE4A	14 vs 16	16740 (1997) vs 13786 (1203)	0.203
PDE4B^a^	14 vs 16	4276 (640) vs 6723 (724)	0.019
PDE4C	14 vs 16	16954 (1458) vs 15439 (1159)	0.418
PDE4D	14 vs 15	8473 (1442) vs 6923 (936)	0.369
PDE5A	14 vs 16	16614 (1514) vs 14736 (1365)	0.363

Only PDE4B elicited significantly altered expression and quantification levels. *P*-values for PDE3A and PDE3B were calculated using Mann–Whitney rank test. All other *p*-values were obtained using the students t-test. A sample from the CRN group was removed for PDE4D after both blinded investigator concluded that quantification was not possible

^a^Marks PDE subtypes with significantly elevated expression levels

## Discussion

### Choice of tissue

Many studies conducted with cancer cell lines have evaluated the importance of PDEs, although most studies only examined one or a few PDE subtypes. The general conclusion from these studies is, yes PDEs are perturbed in transformed cells or tissues [27, 28].

Of note, to our knowledge, there are no studies on the importance of PDE subtypes in non-neoplastic colonic mucosa obtained from patient with CRN.

Our study of PDEs provides support for a CRN predisposition with both perturbed activities of PDE4 and of expression of PDE4B and PDE5A subtypes likely involved in regulatory functions of normal colonic mucosa.

### PDE families selected

Based on a review of the literature, we selected 3 families of the PDE superfamily, PDE3, PDE4, and PDE5 to be evaluated. For members of each of these PDE families, 3 aspects were studied – their activity, although indirectly by SCC, expression by employing qPCR, and tissue localization by immunohistochemistry. Whilst collecting data for our study a publication by Li N et al., Oncogene, reported elevated expression levels of PDE10A in both colon tumor cell lines and clinical tumor specimens. Therefore we conducted an additional q-PCR run examining expression levels of PDE10A in our normal appearing colonic mucosa from patients with CRN and compared it to CTRL. Expression levels of all the remaining PDE subtypes must await later studies, but is relevant to examine to fully asses the role of the entire PDE-family.

### SCC as a measure of PDE activity

Two key intracellular signaling molecules in enterocytes are cAMP and cGMP [29] and their activity is dependent on cNT-PDE activity. Using the SCC as a measure of PDEs activity is based on the knowledge that these enzymes hydrolyze both cAMP and cGMP, and that both these two second messengers are intracellular inducers of anion secretion in colonic mucosa as shown in earlier studies [21], (Fig. 3). Therefore, in normal tissue from patients with and without CRN, PDE function can be measured indirectly by the size of recorded SCC sensitive to PDE inhibitor drugs.

Abnormal levels of either cAMP or cGMP are shown to play a potentially pivotal role in initiating cancer development and for its progression or abrogation [3, 4, 30–32]. PDE enzymes are important regulators of the intracellular level of the mentioned cNTs, as their hydrolysis by PDEs by far exceeds their non-stimulated synthesis. Thus for instance, under basal conditions the intracellular concentration of cAMP is typically kept at less than 5 pmol per mg protein [33].

### Sensitivity of PDE activity, expression and localization

Although it may be argued that use of enzymatic assays and Western blots would be more direct approaches to measurements of PDE activity and abundance than SCC and qPCR, the latter methods carry advantages. Both SCC and qPCR are by far more sensitive and yield much higher accuracy than enzymatic assays and Western blots. For instance, the SCC technique has a sensitivity of measuring changes in ion movement in the range of pmol per second, equal to changes of 0.1 μampere. No ELISA kit can match that. Moreover, the SCC is directly related to the PDE activity located in epithelial cells, elegantly circumventing activity in other locations of the studied tissue. Immunohistochemistry has an advantage over Western blotting by the visualizing location of specific proteins, sometimes even revealing subcellular positions, while often with a near equal sensitivity in the abundance of studied proteins compared with Western blots.

### Levels of PDE activity

Rolipram is a selective inhibitor of PDE4 family members. The response of SCC to rolipram was significantly larger in biopsies from CTRLs compared to CRNs. As PDE4 enzymes primarily hydrolyze cAMP, the result of rolipram-sensitive SCC indicates a reduced elimination of cAMP and reduced control with this signaling pathway in normal colonic mucosa from CRN patients.

The SCC response to cilostamide showed no difference in activity of the PDE3 family of enzymes in non-neoplastic appearing colonic mucosa between patient groups. Meanwhile, the change in SCC due to cilostamide as an inhibitor was on average three times larger than that of rolipram, see results. The balancing control effect of cellular PDE on cNT signaling is thus dominated by PDE3 family members.

Surprisingly, we did not find any SCC-sensitive activity of PDE5 in the biopsy material, despite applying high concentrations of potent inhibitors. An explanation for this lack of activity is low levels of basal intracellular cGMP concentration and failure to introduce significant amounts of cellular cGMP by our basolateral exogenous application of cGMP. Furthermore, applied PDE5 inhibitors sildenafil and zaprinast might not be inhibitors of PDE5 in whole human colonic epithelia tissue. In cell line studies, zaprinast was found not to be a specific PDE5 inhibitor, while specific effects of rolipram, a PDE4 inhibitor, were detected [28]. In contrast to our findings, Li et al. presented a study in which sulindac sulfide was able to inhibit cGMP PDE, including PDE5, in cancer cell lines [34]. The same study also documented inhibitory effects of sildenafil on colon tumor cells. However sulindac sulfide is not an effective inhibitor of colonic PDE5 in whole biopsies and could not be used in our study setting according to manufacturer. So far, the functional role of PDE5 in the development of CRN in man remains unresolved and further studies are warranted.

### Expression of PDEs

Our expressional study detected PDE subtypes 3A, 3B, 4A, 4B, 4C, 4D, 5A and 10A, with PDE3A as the dominant PDE isoform in normal colonica mucosa in both CRN patients and CTRL patients. Nucleotide expression data from a cancer cell line published by Tsukahara et al. suggests PDE3B, and not PDE3A as the main PDE isoform [35]. This is in direct contrast to our findings. In their study, they did not detect any mRNA for PDE3A. Several possible explanations exist for this apparent discrepancy, such as the employed human cancer cell lines did not express PDE3A. This PDE3A and PDE3B discrepancy is another good argument for studying tissue from patients and not just cell lines. A study by Li N. et al. found elevated expression levels of PDE10A in colon tumor cell lines and clinical specimens from colon tumors [36]. We did an additional q-PCR run to examine if this was the case in normal colonic mucosa as well. The same primer was used as in the original study; however, there were no significant differences in expression levels between CRN and CTRL patients. This indicates that expression levels of PDE10A rise when a macroscopic change is seen in the colonic mucosa.

We detected a significantly higher expression of PDE4B in the CRN patients, which has not been previously described in man. Meanwhile, our data on rolipram-sensitive SCC clearly show a lower activity of PDE isozymes in biopsies from CRN patients (Fig. 2), while data on PDE4B from neighboring tissues in the same CRN patients point to both an increased expression (Fig. 4), and an augmented abundance of protein (Fig. 5). Thus, the lower activity of PDE4 detected in functional experiments on CRN-biopsies contradicts the observations of increased tissue expression and abundance of PDE4B: implicating a higher activity of PDE4s. However, there is a simple and likely explanation for the discrepancy between functional and expressional data. The CRN disease might produce a non-functional PDE4B protein with a disease-induced frugal compensatory elevation of its mRNA and protein. This also corroborates the observed higher basal SCC in CRN-biopsies, see Results. Of course, this explanation calls for a future closer look, in which a combination of transcription, translation and post-translation processing of PDE4B and its function is required. A lesson from this part of our study is that determination of protein expression ought to be combined with protein processing and function.

The literature describes PDE5A as overexpressed in breast and colon tumor cells while the expression of other cGMP PDE isozymes is decreased [3, 37]. Our study confirmed that expression levels of PDE5A are increased in CRN patients, which previous studies describe in various cancer cell lines [37, 38]. Our primers were specifically designed to detect mRNA in colon epithelia. We did not take into account splicing and genetic variants of respective PDEs and therefore further studying of these is required. In general, PDE isozymes and their expression in normal colonic tissue is not well described in the literature [39].

### Immunohistochemical localization and quantification of PDEs

Immunohistochemical analysis confirmed that our qPCR was performed on epithelial cells specimens as no underlying nerve and muscle tissue were detected.

Before the start of our study we conducted a search of the literature, which indicated that immunostaining for PDE3A-B, PDE4A-B-C-D and PDE5A often lacked images demonstrating the precision of the antibodies and with very few reports on the topic. We used several of the most recommended antibodies for each PDE. In our material/methods and result sections we present the best antibodies of those employed after consulting with several manufacturers of the PDE antibodies.

We found that antibodies for PDE3A especially and most of the used antibodies were not specifically localized to colonic epithelial cells as underlying connective tissue cells showed high immunoreactivity as well. This result, therefore, demands further research for a better antibody for PDEs in human colonic epithelia.

In regards to PDE5A our findings suggest that it can be seen in normal appearing epithelia while most studies so far have only observed PDE5 in smooth musculature in normal tissue [40]. One study shows that no signaling was observed in the healthy colonic epithelium. However, it was present in cancerous tissue aspiring from colonic epithelia [3]. Further studies are therefore required before it can be concluded that PDE5 is found in normal colonic epithelia.

During our immunohistochemical studies, we employed manual microscopic quantification to assess the abundance of PDEs as a surrogate marker for protein abundance in biopsies. The technique has some drawbacks as described in the literature [41]. Most of the disadvantages are due to inter-observer differences. We attempted to overcome this by blinding two investigators and standardizing assessment protocols. In spite of taking the abovementioned reservations into account the results from the quantification provide an incentive for further investigation into the abundance of PDEs in colonic tissue from patients with CRN and to validate the IHC results. A future investigative modality could be Western blot to verify our finding.

Furthermore, the abundance of PDE5A was lower in CRN compared to controls, but this result was not significant (Table 1.) In contrast q-PCR data showed an elevated level of PDE5A mRNA. Since the result was not significant for the immunohistochemical abundance quantification there is not much to conclude from this. However, if the results described were significant the discrepancy could be attributed to e.g. perturbed translation of the PDE5A mRNA resulting in lower levels of PDE5A enzyme or that produced protein is malformed and therefore not functioning optimally.

## Conclusion

In light of recent reports in the neoplastic cell, we aimed to outline the role of the most prominent PDEs in normal appearing colonic mucosa from patients with CRN. We sought to investigate the function, expression and localization of PDE subtype 3, 4 and 5 in biopsies. PDE10 was investigated with qPCR only. Our data suggests that PDE4B activity is not only dysregulated in CRN per se but also dysregulated in normal appearing mucosa of patients with CRN. In specific PDE4 subtypes could potentially serve as promising pharmacological targets for diagnostic and therapeutic interventions for CRN, although a robust data-driven scientific rationale for selecting PDE4 or any other subtype remains to be challenged and explored accordingly. Furthermore, expression levels of PDE5A are elevated in normal appearing colonic mucosa from patients with CRN, however no differences were observed for this PDE with regards to activity or protein abundance.

## Additional file

Additional file 1: qPCR data for PDE10A. (XLSX 44 kb)

## Abbreviations

AMP: Adenosine monophosphate
cAMP: Cyclic adenosine monophosphate
cGMP: Cyclic guanosine monophosphate
cNTs: Cyclic nucleotides
COX: Cyclooxygenase enzyme
CRC: Colorectal cancer
CRN: Colorectal neoplasia
CTRL: Control
db-cAMP: Dibuturyl cyclic adenosine monophosphate
GMP: Guanosine monophosphate
mRNA: Messenger ribonucleic acid
MUAS: Mini-Ussing air-suction
NSAIDs: Nonsteroidal anti-inflammatory drugs
PDEs: Phosphodiesterases
rt-qPCR: Real-time quantitative polymerase chain reaction
SCC: Short circuit current
SEM: Standard error of the mean
